# Chiloscyphenol A derived from Chinese liverworts exerts fungicidal action by eliciting both mitochondrial dysfunction and plasma membrane destruction

**DOI:** 10.1038/s41598-017-18717-9

**Published:** 2018-01-10

**Authors:** Sha Zheng, Wenqiang Chang, Ming Zhang, Hongzhuo Shi, Hongxiang Lou

**Affiliations:** Department of Natural Product Chemistry, Key Lab of Chemical Biology of Ministry of Education, Shandong University, No. 44 West Wenhua Road, Jinan City, Shandong Province China

## Abstract

This study aimed to characterize the antifungal effects of chiloscyphenol A (CA), a natural small molecule isolated from Chinese liverworts, and investigate its mode of action. CA was effective against five tested *Candida* species with a minimal inhibitory concentration (MIC) of 8–32 μg/ml and exhibited fungicidal activity against *Candida albicans* in both the planktonic state and mature biofilms. The *in vivo* study using *Caenorhabditis elegans* showed that CA prolonged the survival of *C. albicans* infected worms. Further investigations revealed that CA resulted in mitochondrial dysfunction as indicated by mtΔψ hyperpolarization, increased ATP production and intracellular ROS accumulation, and aggregated distribution of Tom70. In addition, CA caused perturbation of the cell membrane and increased membrane permeability, as demonstrated by specific staining and confocal microscopic and transmission electron microscopy (TEM) observations and by calcein-leakage measurements. This conclusion was further confirmed by the decreased cell size of CA-treated cells via three-dimensional contour-plot analysis using flow cytometry. Taken together, these results suggest that CA exerts fungicidal activity by eliciting both mitochondrial dysfunction and plasma membrane destruction in *C. albicans*. The elucidated mechanism supports the potential application of CA against clinical fungal infections.

## Introduction

The incidence of fungal infections has increased at an alarming rate in recent decades^1–3^. *Candida* species are predominant nosocomial fungal pathogens that threaten immunocompromised and high-risk surgical patients, causing high morbidity and mortality^1,4^. Among them, *Candida albicans* is the most common cause of invasive infections and is responsible for more than 50% of human candidiasis, including superficial infections and systemic infections, with a mortality of approximately 40%^4–7^.

Large-scale application of broad-spectrum antifungal agents and the introduction of protocols for antifungal prophylaxis in patients at risk have increased the prevalence of drug resistance^8,9^. In addition, antifungal drugs available for the treatment of systemic fungal infections are limited^10,11^. Therefore, the development of novel and more effective antifungal therapeutics is of paramount importance.

Plant products have been used as traditional medicines and as a major resource for drug discovery^12,13^. In plants, phytochemicals often act as protective agents against external stress and pathogenic invasion^14^, and some plant phytochemicals possess antimicrobial effects^15^. Liverworts are simple nonvascular plants that are rich sources of terpenoids, aromatic compounds and acetogenins with diverse biological activities, including cytotoxic, anti-oxidative, insect antifeedant, antimicrobial and antifungal activities^16^. As part of our ongoing research on biologically active natural products from two Chinese liverworts, *Chiloscyphus polyanthus* (L.) and *Bazzania albifolia* Horik., we obtained chiloscyphenol A (CA), a sesquiterpenoid with a novel rearranged calamenene skeleton^17,18^. In our preliminary study, CA exhibited potent antifungal activity against *C. albicans* ^18^. The aim of the present study was to characterize the antifungal activity of CA against various *Candida* species and elucidate its mode of action. We determined that the antifungal mechanisms of CA in *C. albicans* include mitochondrial dysfunction and destruction of the cytoplasmic membrane.

## Results

### The antifungal effects of CA on *Candida* species

**Figure 1:**
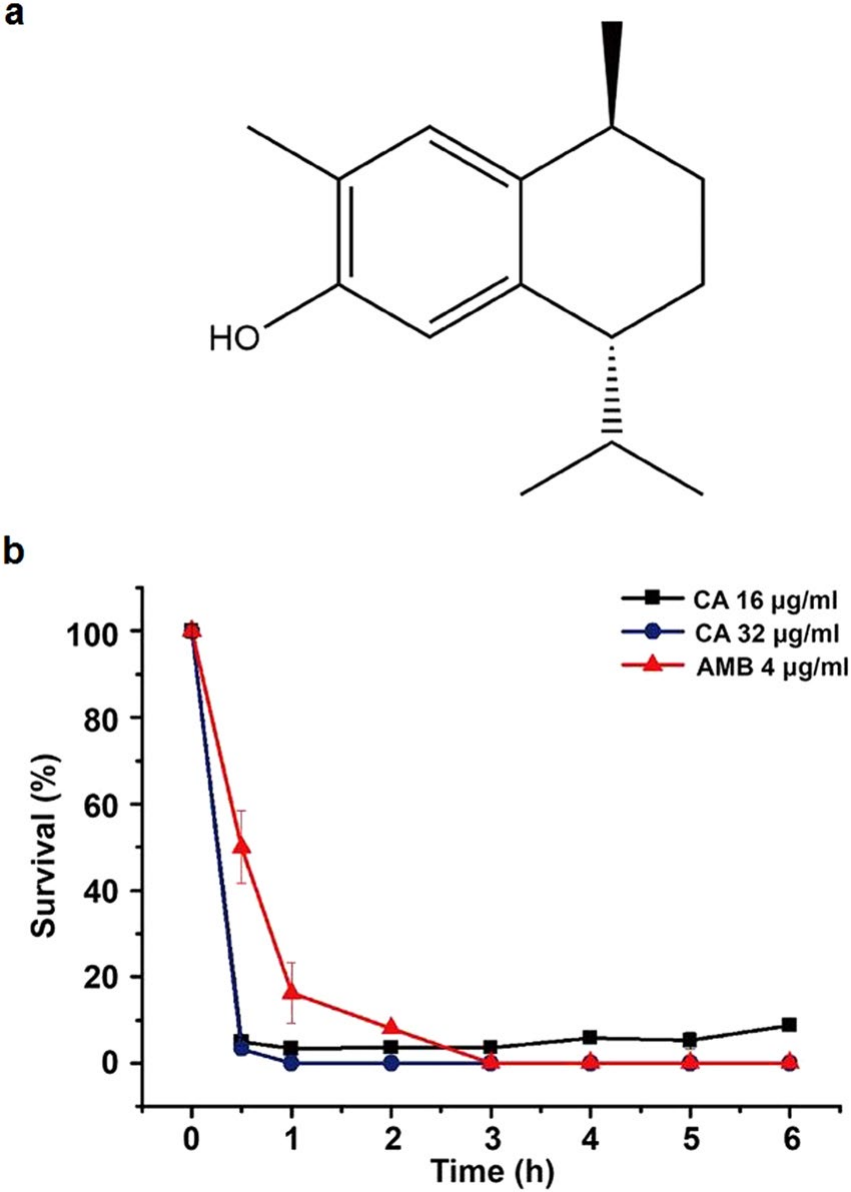
Time-killing kinetics of CA and AMB against *C. albicans*. (**a**) Chemical structure of CA. (**b**) *C. albicans* SC5314 was treated with CA or AMB (positive control) for 3 h. The survival rates were then calculated based on colony survival. The results are shown as the averages and standard deviations from three independent experiments.

We determined the minimal inhibitory concentration (MIC_80_) of CA against *C. albicans* strains, including the wild-type strain SC5314 and clinical isolates comprised of azole-resistant and azole-susceptible strains. The antifungal activity of CA was also assessed against other clinically derived *Candida* species: *Candida krusei*, *Candida tropicalis, Candida glabrata* and *Candida parapsilosis*. As shown in Table 1, CA exhibited potent antifungal activity against these five tested *Candida* species, with MICs ranging from 8 to 32 μg/ml. In the time-killing assay, 16 μg/ml CA or greater eliminated 90% of planktonic *C. albicans* cells within 1 h, an efficacy comparable to that of 4 μg/ml Amphotericin B (AMB) after 3 h of treatment (Fig. 1b). This result indicated that CA possesses a quick mode of fungicidal action against *C. albicans*.

**Table 1:** The antifungal activity of CA with AMB as the positive control.

Strains	MIC_80_ (μg/ml)		
	CA	AMB	FLC^a^
SC5314 (*C. albicans*)	16	0.5	2
11D (*C. albicans*)	8	0.5	2
23E (*C. albicans*)	16	0.5	4
CA1 (*C. albicans*)	16	0.5	<2
148 (*C. albicans*)	16	0.5	<2
162 (*C. albicans*)	16	0.5	<2
28 A (*C. albicans*)	16	0.5	>128
28D (*C. albicans*)	16	0.5	>128
28I (*C. albicans*)	16	0.5	>128
28 J (*C. albicans*)	16	0.5	>128
CK1 (*C. krusei*)	32	0.5	2
CG1 (*C. glabrata*)	16	0.5	2
CT1(*C. tropicalis*)	8	0.5	2
CT3(*C. tropicalis*)	32	1	>128
CP1((*C. parapsilosis*)	32	0.5	2

^a^The MICs values of FLC have been reported in our previous publications.

### CA inhibits *C. albicans* biofilm formation

**Figure 2:**
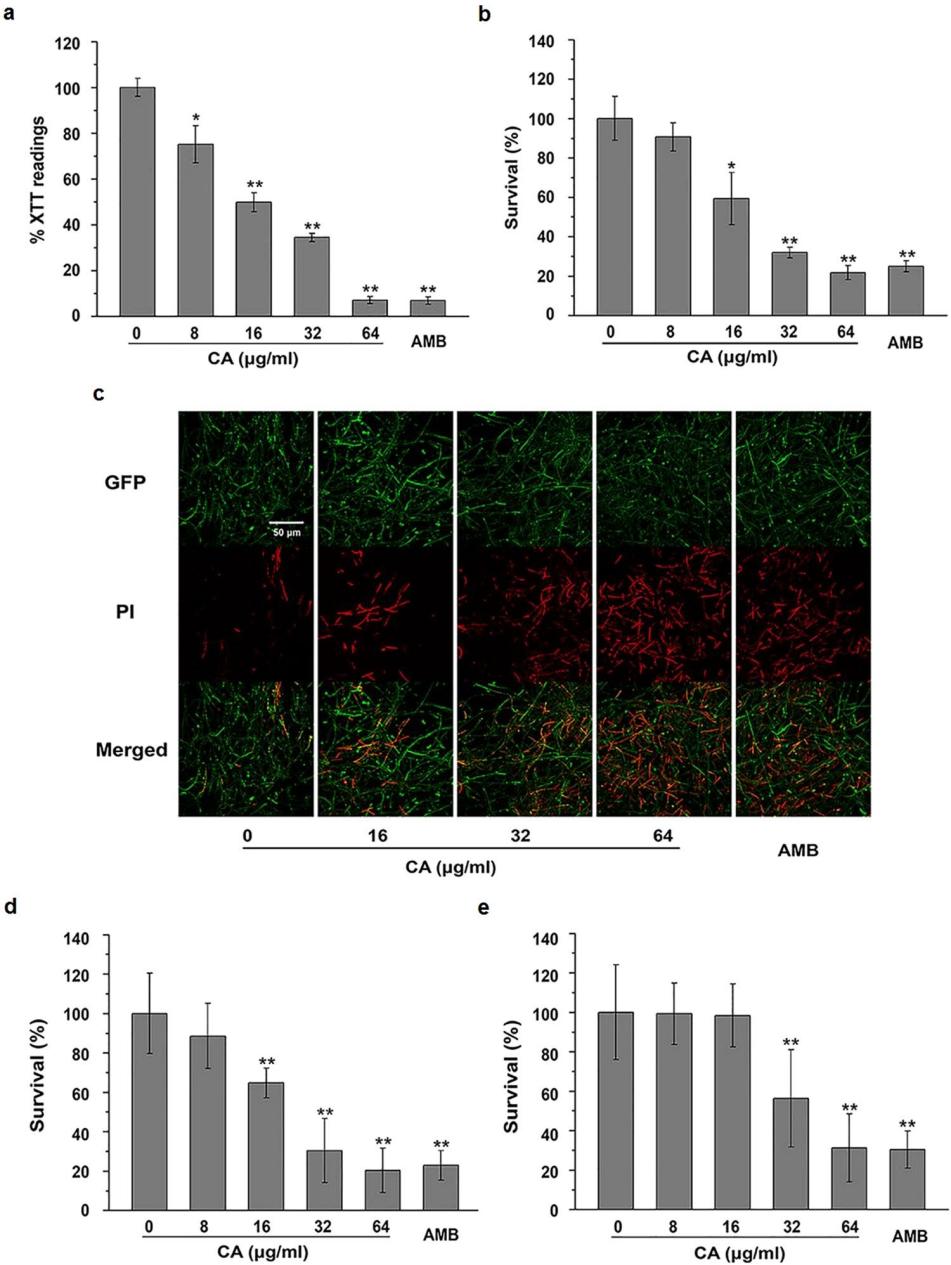
The antifungal effect of CA on *Candida* biofilms. (**a**) The effect of CA on wild-type *C. albicans* biofilm formation was assessed by the XTT reduction assay. (**b**) The fungicidal effect of CA on mature biofilms of SC5314 was estimated by the total viable counts. (**c**) Mature biofilms of *C. albicans TDH3*-*GFP*-CAI4 were stained with PI after 24 h of treatment with CA and visualized by CLSM. The eradicating effects of CA on mature biofilms of FLC-resistant *C. albicans* 28 A (**d**) and *Candida tropicalis* CT3 (**e**) were assessed by calculating cell survival. The bars represent the means ± SD values of three independent experiments. *P < 0.05, **P < 0.01.

Most microbial infections involve biofilm formation, which increases the risk of relapse of chronic infection^19^. *C. albicans* biofilms are a highly organized community of cells and form a unique architecture that is not easily eradicated using current therapy methods^20^. *Candida* biofilm formation is a virulence factor in candidiasis not only due to the increased resistance of biofilms to antifungal agents but also the ability of biofilms to withstand host immune defenses^21^. Thus, inhibition of biofilm formation or eradication of established biofilms is an attractive approach in antifungal therapy. To provide a more quantitative assessment of the inhibitory effect of CA on *C. albicans* biofilms, an XTT reduction assay was performed to measure the biofilm biomass as previously described^22^. CA treatment resulted in 50.11% ± 4.10% inhibition of biofilm growth at a dose of 16 μg/ml. When the dose was increased to 64 μg/ml, greater than 90% inhibition of biofilm growth was observed (Fig. 2a).

### CA displays fungicidal activity against preformed *Candida* biofilms

In addition to its inhibitory effect on biofilm formation, CA exhibited fungicidal effects on preformed mature biofilms of *C. albicans*. Comparing with the drug-free group, 16 μg/ml CA (one-fold MIC dose) resulted in approximately 40% cell death within mature biofilms. When the dose increased to 32 or 64 μg/ml (two-fold or four-fold MIC dose), CA resulted in greater than 60% killing within mature biofilms (Fig. 2b). These findings were visually confirmed by confocal laser scanning microscope (CLSM) inspection (Fig. 2c).

In addition to the wild-type strain, CA exhibited biofilm-eradicating activity against fluconazole (FLC)-resistant *C. albicans* 28 A (Fig. 2d). The biofilm-eradicating ability of CA was also verified in other *Candida* species, such as FLC-resistant *C. tropicalis* CT3 (Fig. 2e).

### CA exhibited antifungal activity *in vivo* using a nematode model

**Figure 3:**
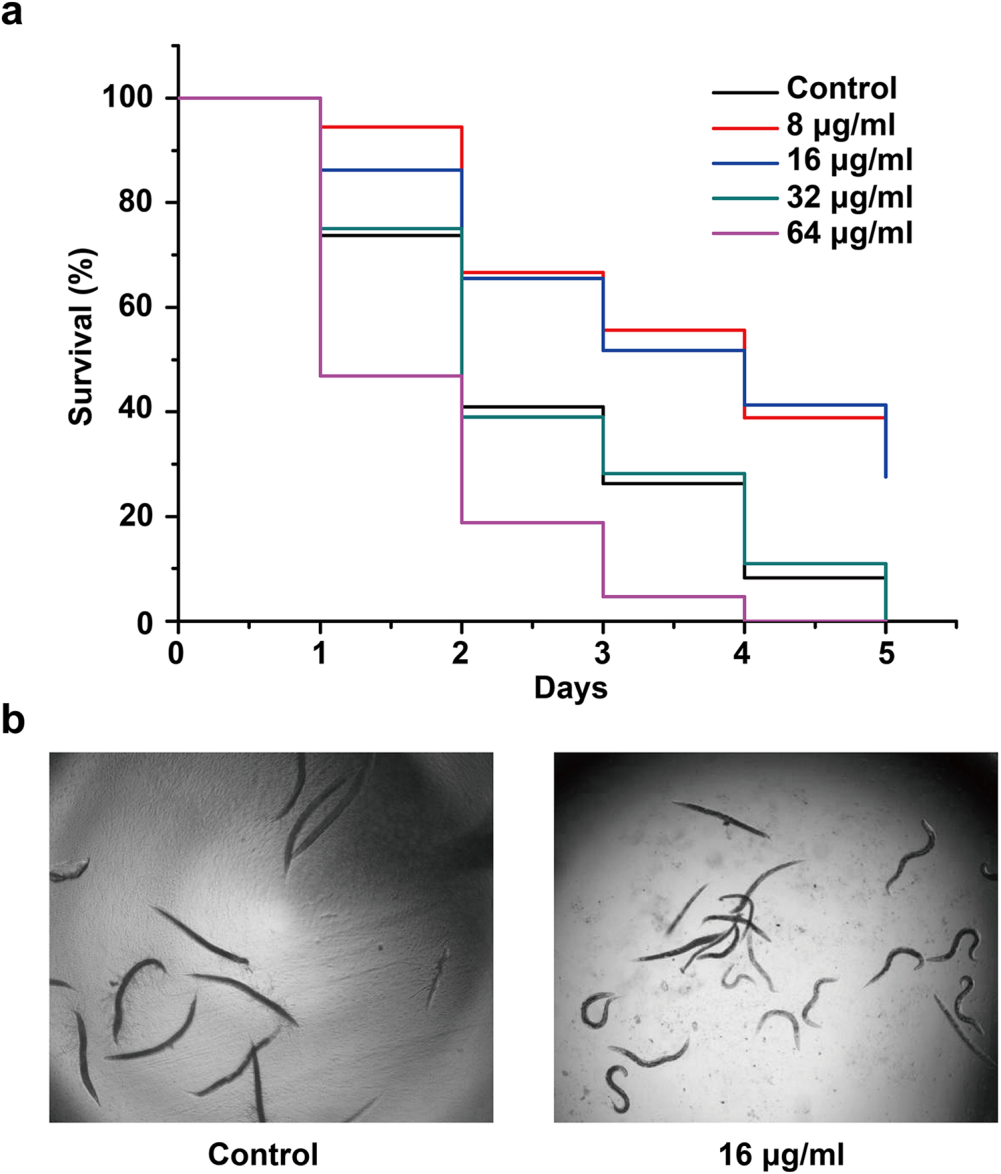
The efficacy and toxicity of CA in treating *C. albicans*-infected *C. elegans*. (**a**) Infected nematodes by *C. albicans* SC5314 were treated with 1% DMSO (negative control) or CA (8, 16, 32 or 64 μg/ml). Each day the worms were monitored and the survival rate was calculated. P value is less than 0.001 for 8 μg/ml and 16 μg/ml of CA treated groups compared with vehicle-treated group. Each group contains about 60 nematodes and this assay was repeated twice. (**b**) The infected nematodes by *C. albicans* in control group or CA-treated group were imaged by a light field microscope.

We then evaluated the efficacy and toxicity of CA using *Caenorhabditis elegans* as an infectious model. In the nematode infection assay, the state of the worms was monitored daily to record dead/live rates. Results showed that CA prolonged the survival of *C. elegans* infected by *C. albicans* at the dose of 8 or 16 μg/ml (Fig. 3a). CA at the dose of 32 µg/ml or above displayed toxicity towards nematodes (Fig. 3a). Microscopic inspection revealed that the worms in the negative control group were infected with an abundance of *C. albican*s hyphae penetrating within their bodies while the worms under CA treatment (16 μg/ml) remained in the curly growth state (Fig. 3b). These data suggested a potential application of CA in treating *C. albicans* infection *in vivo*.

### CA induces ROS production

**Figure 4:**
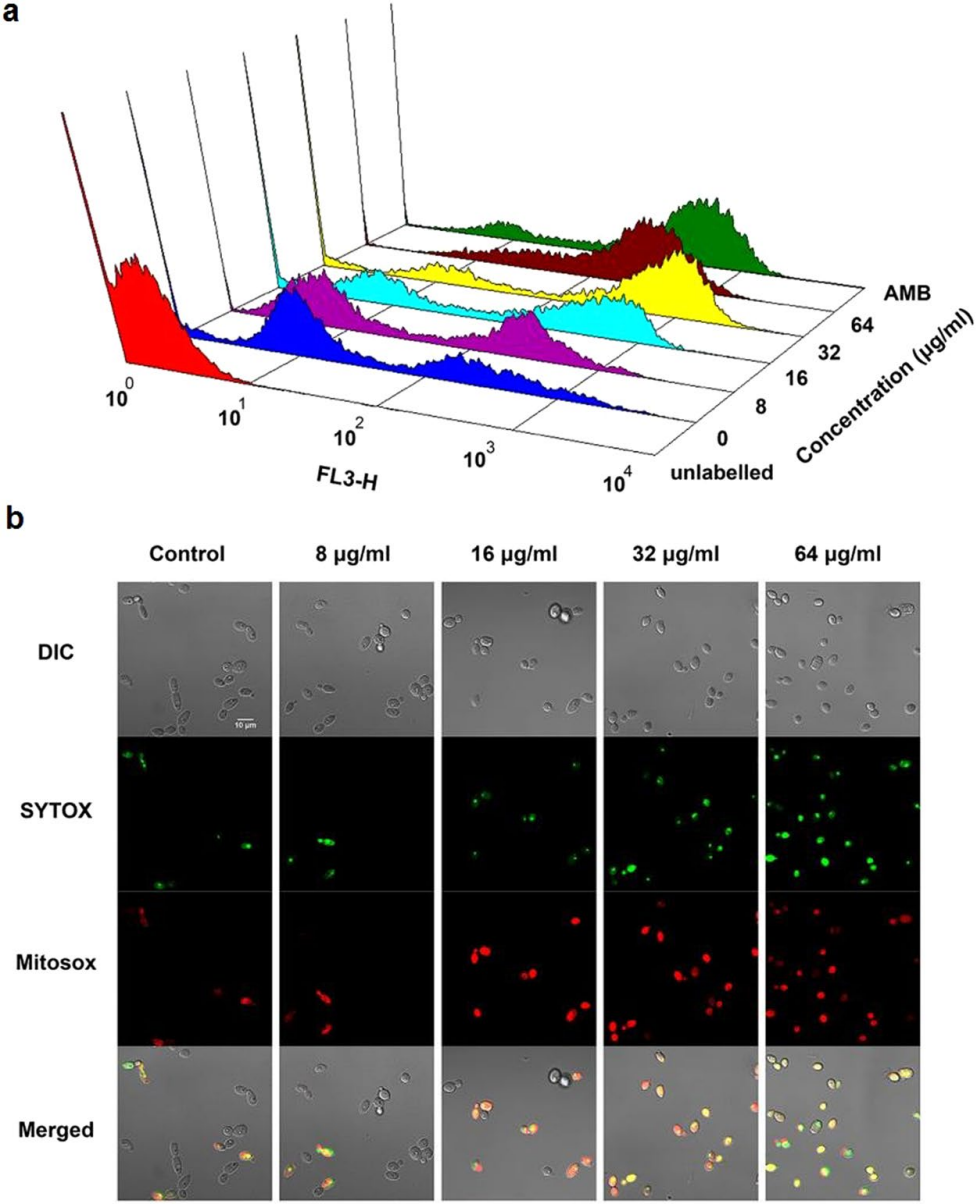
Effect of CA on intracellular ROS generation. SC5314 cells were exposed to CA or AMB (positive control) for 3 h. After staining with MitoSOX Red (**a**) or a combination of MitoSOX Red and SYTOX Green (**b**), the samples were analyzed by flow cytometry (**a**) or visualized by CLSM (**b**).

**Figure 5:**
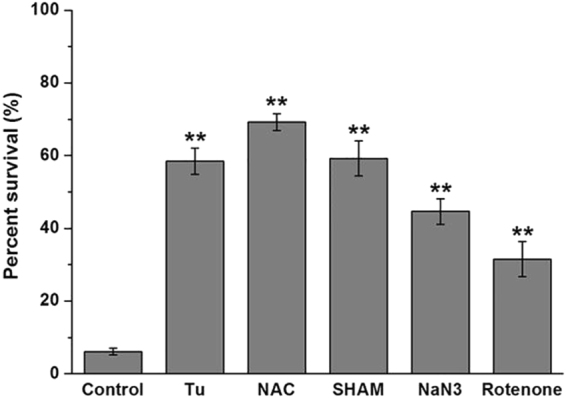
Effect of antioxidents and respiratory chain inhibitors on the susceptibility of *C. albicans* to CA killing. *C. albicans* cells preincubated with or without antioxidents (Tu, NAC) or respiratory chain inhibitors (SHAM, NaN3 or rotenone) were exposed to 16 μg/ml CA. The viability of the cells was assessed by colony counting method. The bars represent the means ± SD values of three independent experiments. **P < 0.01.

Reactive oxygen species (ROS) are byproducts of cellular metabolism and are primarily generated in the mitochondria. The formation of excessive ROS is likely to cause cell damage and has been considered as a fungicidal mechanism^23^. The fluorescent probe MitoSOX was utilized as a ROS indicator to determine the amounts of ROS generated when *C. albicans* was exposed to CA. Flow cytometry analysis demonstrated that CA promoted intracellular ROS generation in *C. albicans* in a dose-dependent manner after 3 h of treatment (Fig. 4a). To elucidate the relationship between cell death and ROS accumulation induced by CA, the treated cells were stained with MitoSOX and SYTOX Green and examined by CLSM. CA-treated cells emitted both green and red fluorescence, as shown in Fig. 4b, indicating that ROS was restricted to dead cells. Furthermore, in the presence of the oxygen scavenger thiourea (Tu) or N-acetyl-L-cysteine (NAC), CA-mediated cell killing was prevented. The survival ratio increased from 6.08% ± 0.93% (control) to 58.46% ± 3.531% (Tu) and 69.23% ± 2.31% (NAC) (Fig. 5). These data suggested that the cell death mediated by CA was at least partially attributable to cumulative intracellular ROS.

**Figure 6:**
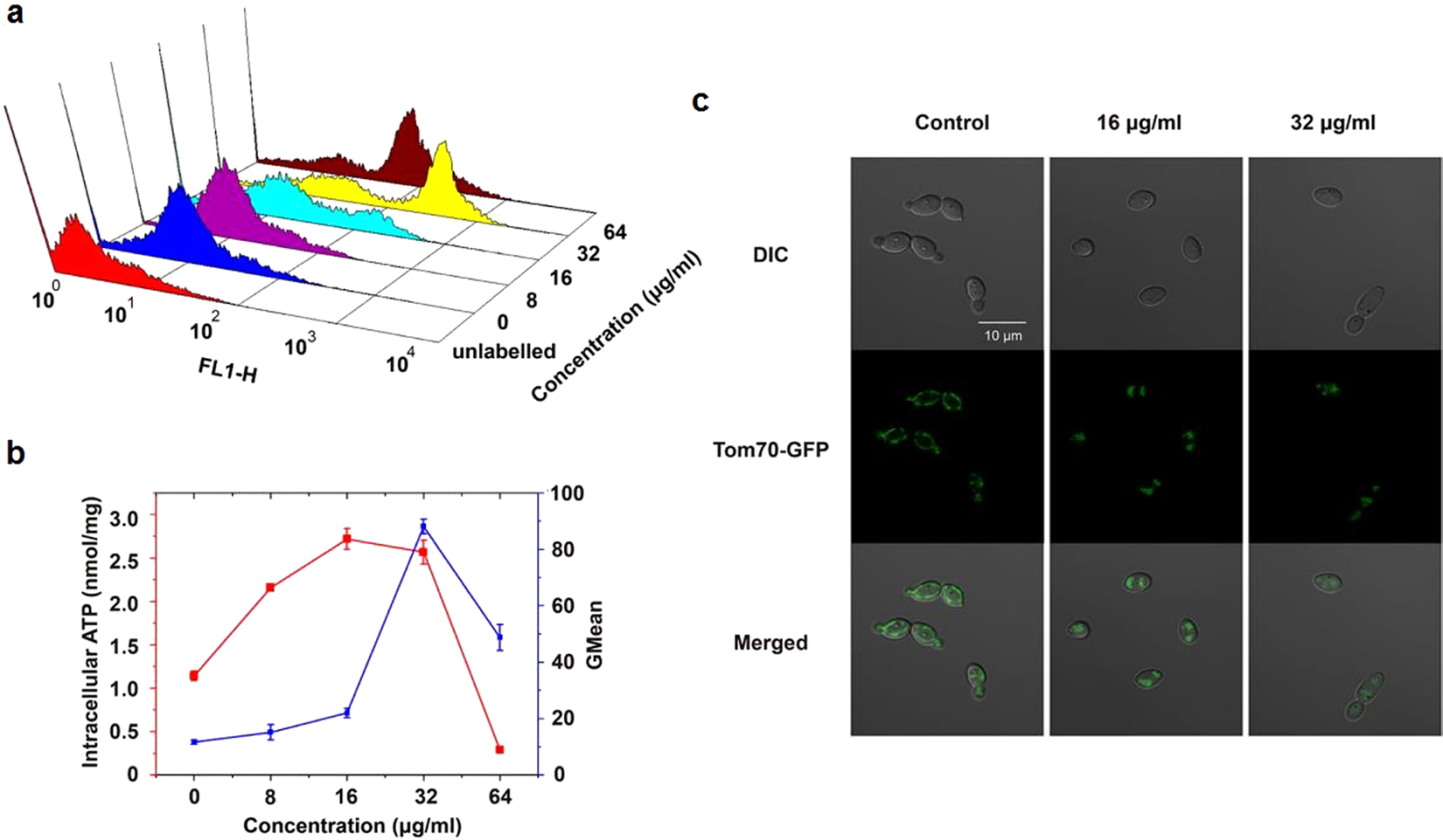
Effect of CA on mitochondrial function. (**a**) SC5314 cells were cultured in SD medium with CA or AMB (positive control) for 3 h before staining with 20 μM Rh123 to indicate alteration of mtΔψ. The fluorescence intensity was analyzed by flow cytometry. (**b**) Quantitative assessment of mtΔψ based on the geometric mean (Gmean) value from flow cytometry analysis in (**a**). The intracellular ATP levels in CA-treated *C. albicans* cells were quantified using an ATP assay kit. (**c**) *C. albicans TOM70*-*GFP*-CAI4 was treated with various concentrations of CA. After 3 h, the cells were visualized by CLSM.

To determine whether the ROS accumulation induced by CA is related with the metabolic state of mitochondria, we analyzed the mitochondrial membrane potential **(**mtΔψ), an indicator of the energetic state of the mitochondria^24^, and intracellular ATP content. Flow cytometry analysis using Rhodamine 123 (Rh123) revealed that CA increased the fraction of cells with high fluorescence intensity (Fig. 6a and b), suggesting increased mtΔψ in the presence of CA. ATP also increased with elevated mtΔψ (Fig. 6b). At a dose of CA of 64 μg/ml, the mtΔψ dramatically decreased, with a corresponding decrease in ATP content (Fig. 6b). Moreover, as shown in Fig. 5, addition of respiratory chain inhibitors such as NaN_3_, salicyl hydroxamic acid (SHAM), or rotenone protected *C. albicans* against the the fungicidal activity of CA. These observations demonstrated that mitochondrial respiration was affected by CA, which led to the production of ROS.

The outer mitochondrial membrane (TOM), a multi-subunit complex, is responsible for the specific recognition and membrane translocation of nuclear-encoded preproteins^25^. Tom70 is a major surface receptor for mitochondrial protein precursors in the TOM complex^26^ and was used as a mitochondrial membrane marker in this study. The green fluorescent protein (GFP) -labeled *C. albicans* strain *TOM70*-*GFP*-CAI4 was previously constructed in our lab and was used to monitor the effect of CA on the mitochondria. CLSM inspection based on Tom70-GFP imaging revealed that mitochondria in non-drug-treated cells displayed a tubular structure, while those in CA-treated cells presented an aggregated distribution (Fig. 6c). These results suggested that CA disrupted the normal function of mitochondria.

Taken together, CA led to altered mitochondrial respiration and stimulated intracellular ROS accumulation in *C. albicans*, ultimately resulting in mitochondrial dysfunction and thus contributed to cell death.

### CA disrupts the integrity of the plasma membrane

**Figure 7:**
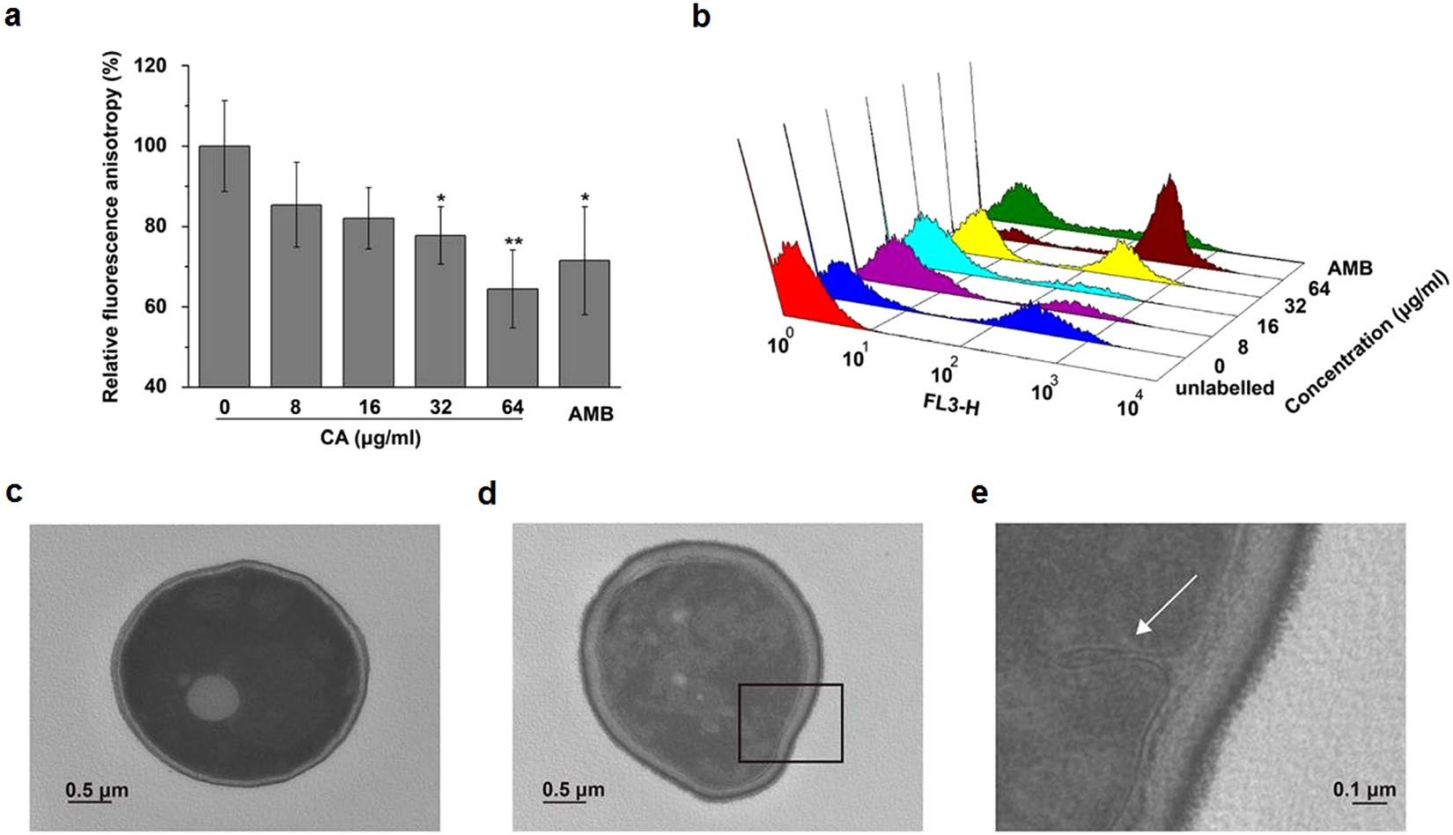
Effect of CA on cell membrane integrity. (**a**) The effect of CA on cell membrane dynamics. SC5314 cells were incubated with various doses of CA or 4 μg/ml AMB (positive control) for 3 h, followed by DPH staining and spectrofluorophotometric detection. The bars represent the means ± SD. (**b**) Increased cytoplasmic membrane permeabilization was observed after *C. albicans* cells were treated with CA or 4 μg/ml AMB (positive control). The treated cells were stained with PI and analyzed by flow cytometry. (**c–e**) Transmission electron micrographs of *C. albicans* treated with or without 32 μg/ml CA. (**c**) Vehicle control group. (**d**) CA-treated group. (E) Enlarged observation of the rectangular area in (**d**).

The integrity of the cell membrane is critical to maintaining a healthy cellular state. To determine if CA affected the normal function of the fungal plasma membrane, the fluorescent stains 1,6-diphenyl-1,3,5-hexatriene (DPH) and propidium iodide (PI) were applied to analyze the dynamics and permeability, respectively, of the cell membrane in *C. albicans*. DPH is a lipophilic fluorescent probe that is commonly used as a reporter of the highly disordered hydrophobic core of the bilayer^27^. The DPH fluorescence intensity in CA-treated cells decreased in a dose-dependent manner, indicating perturbation of the membrane lipid bilayer under CA treatment (Fig. 7a). PI is a membrane-impermeable nucleic acid fluorescent stain that penetrates damaged or permeabilized cell membranes and emits red fluorescence^28^. Flow cytometry (Fig. 7b) revealed that CA increased cell membrane permeabilization in a dose-dependent manner. In the transmission electron microscopy (TEM) micrographs, untreated cells showed normal cellular morphology with a distinct cell membrane (Fig. 7c), whereas the plasma membrane was destroyed in CA-treated cells (Fig. 7d). Specifically, CA resulted in loss of the elasticity of the cytoplasmic membrane and peeling of the membrane from the cell wall (Fig. 7d and e). In certain local areas, the cytoplasmic membrane curled inward (indicated by the arrow) under CA stress (Fig. 7e). Collectively, these results suggested that CA disrupted the cell membrane, thus contributing to the cell death of *C. albicans*.

### Morphological changes induced by CA in *C. albicans*

**Figure 8:**
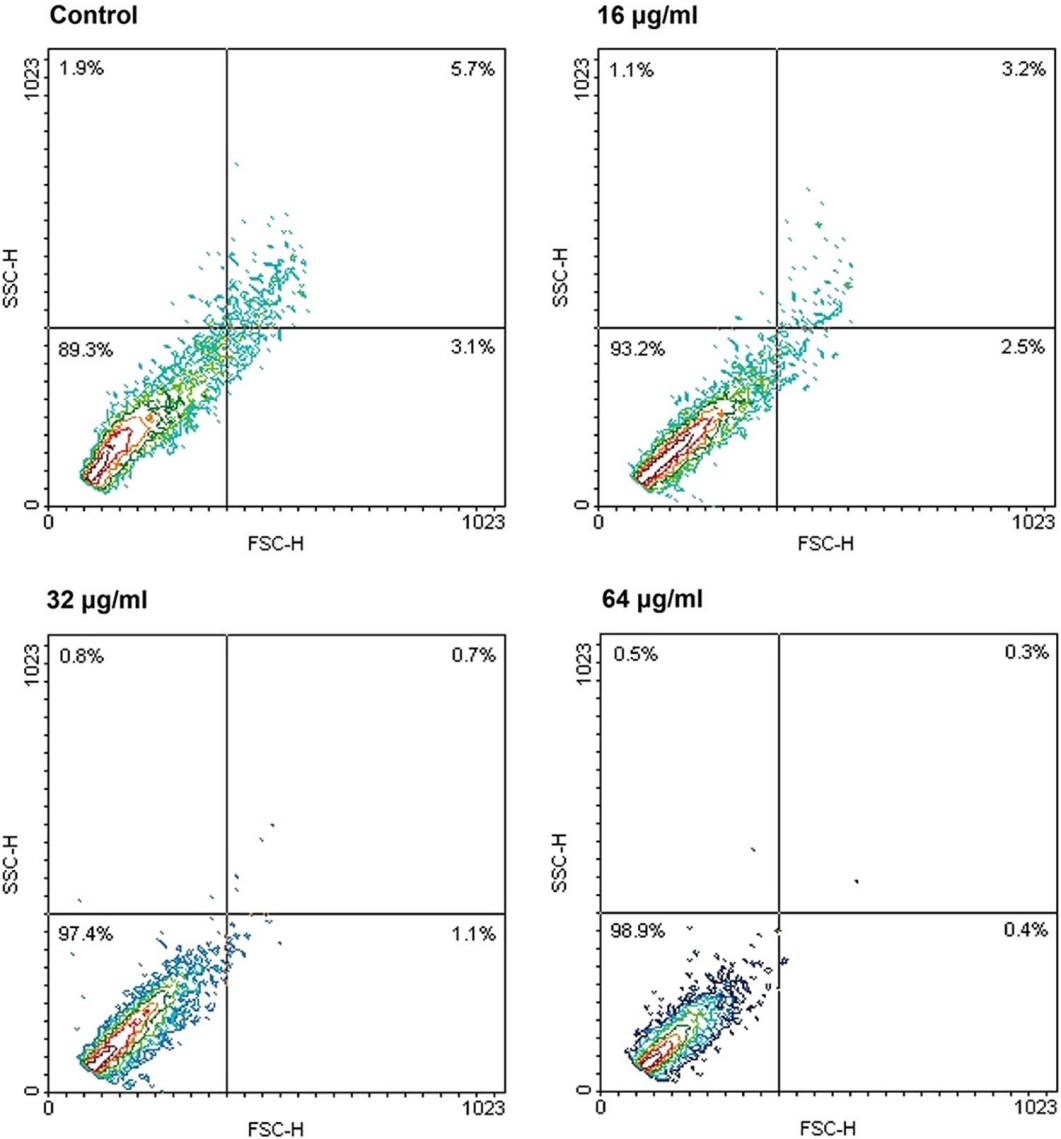
Morphological changes induced by CA. The alterations of cell morphology were analyzed by flow cytometry. FSC (y-axis) is an indicator of size, SSC (x-axis) is an indicator of granularity.

The effects of CA on the morphology of *C. albicans* cells were further examined by flow cytometry using forward scatter (FSC) as an indicator of size and side scatter (SSC) as an indicator of granularity. CA treatment shifted the *C. albicans* population to an area with low FSC and SSC values compared with the control group (Fig. 8), implying that CA treatment resulted in cell shrinkage.

### The disruptive effect of CA on an artificial membrane

**Figure 9:**
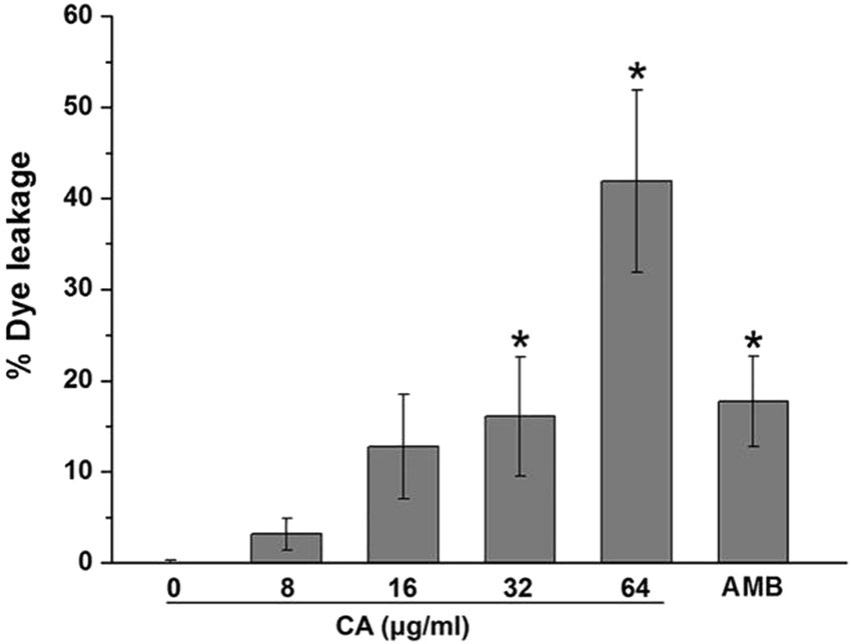
The disruptive effect of CA on an artificial membrane. Calcein-entrapped LUVs composed of phosphatidylcholine/phosphatidylethanolamine/phosphatidylinositol/ergosterol (5:4:1:2, w/w/w/w) were treated with CA or AMB, and the fluorescence intensity of the released calcein was measured by a fluorescence microplate reader. The bars represent the means ± SD. *P < 0.05, **P < 0.01.

To further verify the disruptive effect of CA on the cell membrane, we performed an *in vitro* study to assess the interaction of CA with an artificial membrane (large unilamellar vesicles, LUVs). When the liposomal membrane was treated with membrane disrupters, calcein was released from the LUVs and emitted green fluorescence. The leakage of entrapped calcein was positively proportional to the CA dose when the LUVs were incubated with CA (Fig. 9). This result suggested that CA indeed destroys the cell membrane and thereby causes cell death.

### The toxicity of CA against human cell lines

**Figure 10:**
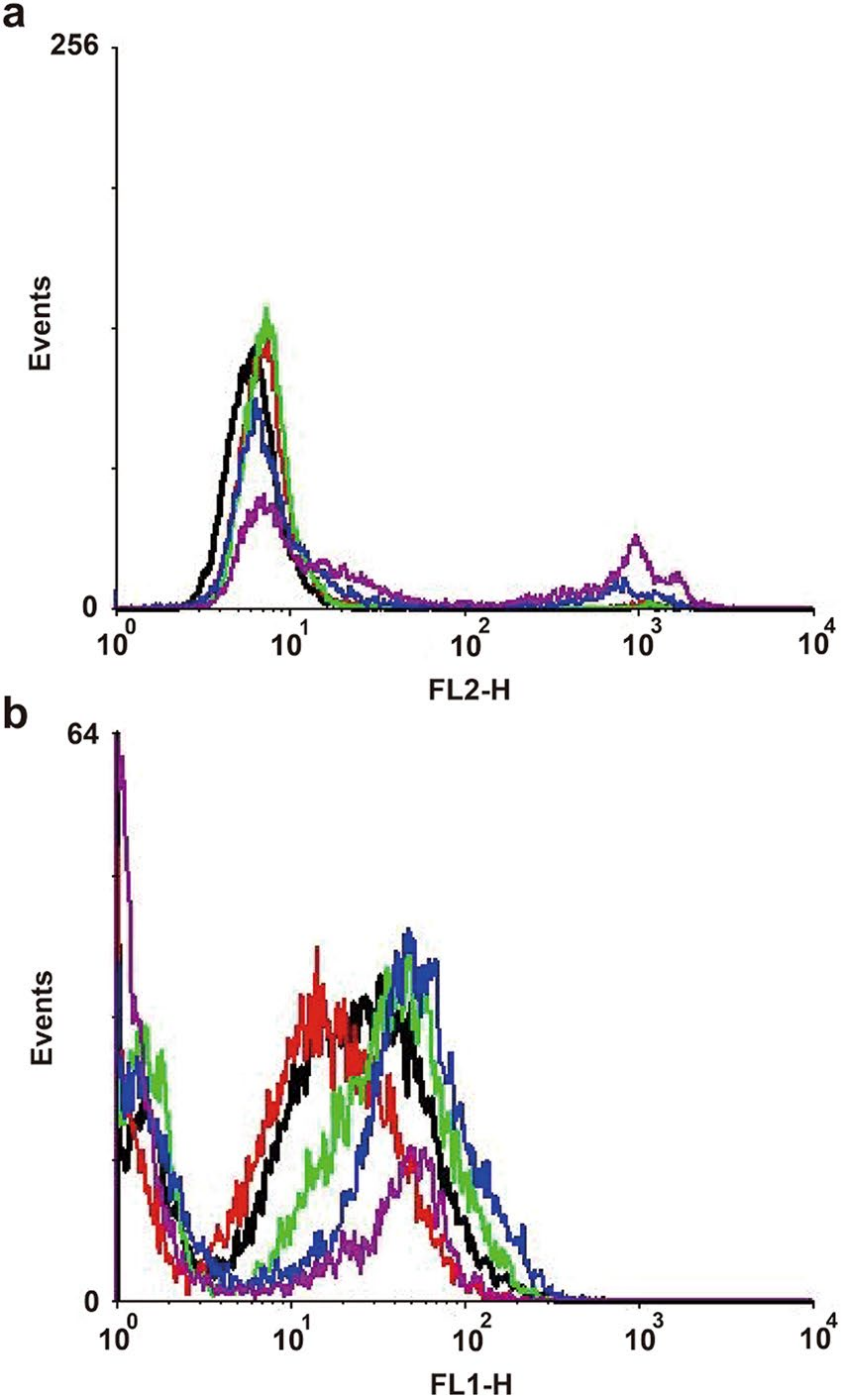
The cytotoxicity of CA. Hela cells were cultured for 24 h, serial concentrations of CA was added into cells. After 12 h treatment, cells were collected and stained with 5 μg/ml of PI or 5 μM DCFH-DA for 30 min in the dark. (**a**) The cell membrane permeability was indicated by the cell penetration of PI, revealed by the flow cytometry measurement. (**b**) The intracellular ROS contents were detected by flow cytometry based on the fluorescence intensity. Black line, control group in which HeLa cells were stained with DCFH-DA; red line, group treated with 4 µg/ml of CA; green line, group treated with 8 µg/ml of CA; blue line, group treated with 16 µg/ml of CA; purple line, group treated with 32 µg/ml of CA.

We utilized normal human bronchial epithelium (HBE) cells and HeLa cells to evaluate the cytotoxicity of CA. The half maximal inhibitory concentrations (IC_50_) of CA against HBE and HeLa cells are 12.78 ± 1.3 μg/ml and 11.16 ± 2.0 μg/ml, respectively. To determine whether CA destroy the cell membrane of human cells, cell membrane impermeable fluorescent dye PI was used to determine the permeability of HeLa cells under the treatment of CA. The results showed that CA did not result in significant PI penetration until the concentration reached 32 μg/ml, suggesting the cytotoxicity of CA at 32 μg/ml or above (Fig. 10a). We also utilized HeLa cells to detect the intracellular ROS formation in the presence of CA. The results showed that CA at concentrations of 8–32 μg/ml just caused a slight increase of ROS generation (Fig. 10b).

## Discussion

With the wide application of antibiotics, the prevalence of resistant strains has reduced the effectiveness of current antifungal agents. In addition, the majority of *C. albicans* infections are associated with biofilm formation^1^. These biofilms exhibit greatly increased resistance to antifungal agents and an enhanced ability to withstand host immune defenses^21^. However, few drugs are effective against drug-resistant strains or mature biofilms, highlighting the importance of developing new therapeutics for evading drug resistance and attacking biofilms.

Natural products either from microorganisms or plants are a rich source of new antifungal agents^29–31^. In this study, CA derived from Chinese liverworts was found to display potent activity against both FLC-susceptible and FLC-resistant *Candida* isolates (Table 1). Notably, maximal activity was reached within 1 h, much more quickly than AMB (Fig. 1b). Moreover, CA displayed potent activity in preventing biofilm formation by *C. albicans* and targeting cells within mature biofilms, including against strains resistant to FLC. The activity against preformed *C. albicans* biofilms is noteworthy because the cells within biofilms exhibit highly resistant characteristics via upregulation of efflux pumps and production of persisters, *etc* ^32^. These observations suggest that CA may be useful for blocking fungal biofilm formation on biomedical device surfaces or for disinfecting those surfaces after an infection has become established. These results prompted us to investigate the mode of action of CA.

We observed that CA treatment induced intracellular ROS generation in *C. albicans* in a dose-dependent manner (Fig. 4a) and the cell death caused by CA was positively correlated with the generated ROS (Fig. 4b). Moreover, the ROS scavengers Tu and NAC could decrease the killing rate of CA (Fig. 5). These results are consistent with previous reports that some agents exert antifungal activity via ROS^33–35^.

Mitochondria is a major source of intracellular ROS. We found that the mtΔψ and ATP levels were greatly elevated in the presence of 8–32 μg/ml CA and sharply decreased when the dose increased to 64 μg/ml (Fig. 6b). Further observation showed that blocking of the mitochondrial respiration by using SHAM, NaN3 or rotenone inhibited the antifungal action of CA to a certain extent (Fig. 5), suggesting that CA probably disturbed the mitochondrial respiration and induced ROS accumulation and ultimately destroyed the normal function of the mitochondria.

DPH and PI are indicators of cell membrane kinetics and permeabilization. Treatment with CA decreased the fluorescence intensity of DPH and markedly increased the number of PI-stained cells, implying that CA treatment damaged the integrity of the cell membrane. TEM showed that CA treatment resulted in a loss of the elasticity of the cytoplasmic membrane and peeling of the plasma membrane from the cell wall (Fig. 7d). The shrinkage of CA-treated cells and disruptive effect of CA on an artificial membrane (Figs 8 and 9) provided further evidence of destruction of the cell membrane induced by CA.

**Figure 11:**
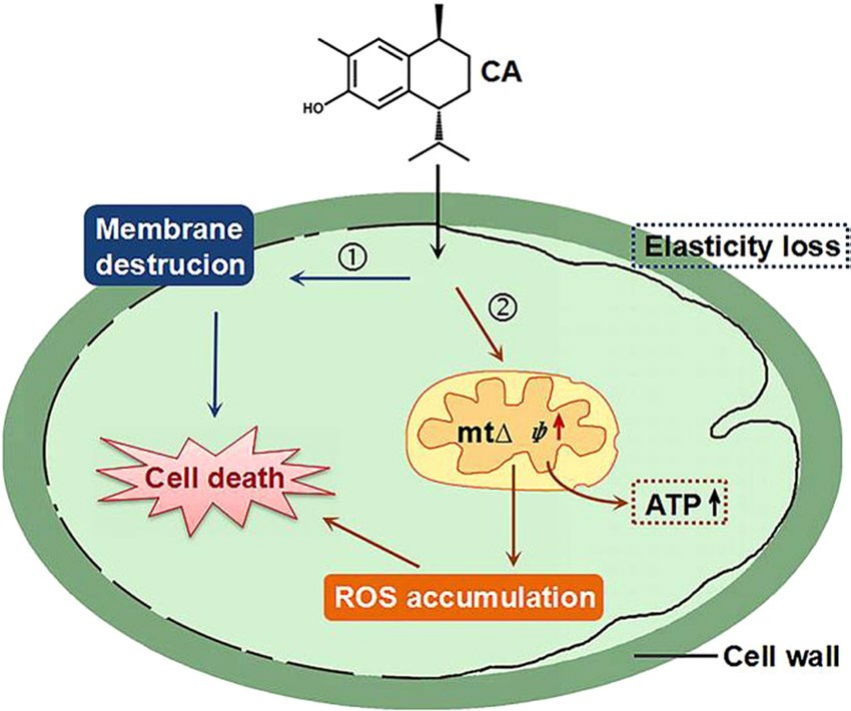
Outline of the potential mechanisms by which CA elicits cell death in *C. albicans*. CA disturbs mitochondrial respiration and induces ROS generation and thus destroys the normal function of the mitochondria. Mitochondrial dysfunction together with the disruption of the cytoplasmic membrane by CA contributes to cell death in *C. albicans*.

Based on these findings, we propose a model to explain the effects of CA on *C. albicans* (Fig. 11). Generally, cell death triggered by CA is likely a consequence of ROS accumulation and cell membrane disruption. These two factors contributing to the *C. albicans* cell death are probably responsible for the toxicity of CA against human cell lines and nematodes (Figs 3 and 10) although the toxicity of CA against HeLa cells is primarily attributed to the damage to cell membrane. Although the low therapeutic index of CA restricted it to be developed as a clinically antifungal drug, the quick fungicidal action and potent activity against resistant strains and mature biofilms were still worth attention. The therapeutic index of CA could be increased by the further chemical modification, as the improved antineoplastic activity of riccardin D-N through aminomethylation of riccardin D in our previous study^36^. This would broaden the potential application of CA.

In nature, organisms have evolved multiple strategies for responding to environmental challenges such as oxidative stress and microbial infections^37–39^. We previously isolated CA from two types of Chinese liverworts, which implies that Chinese liverworts produce protective substances such as CA to resist fungal infections.

In summary, CA acts as a potent fungicidal agent, which expands the current potential antifungal agents, although the cytotoxicity of CA is not particularly restricted to *Candida* pathogens. The elucidation of the antifungal mechanism of CA provides insights into the modes of action of other sesquiterpenoids as well as intrinsic mechanisms of liverworts in resisting microbial invasion.

## Materials and Methods

### Strains and culture conditions

The wild-type *C. albicans* strain SC5314, nine clinical isolates of *C. albicans* (11D, 23E, CA1, 148, 162, 28 A, 28D, 28I, and 28 J), two GFP-tagged *C. albicans* strains (*TDH3*-*GFP*-CAI4 and *TOM70*-*GFP*-CAI4), and clinical isolates of *C. krusei* (CK1), *C. glabrata* (CG1), *C. tropicalis* (CT1, CT3) and *C. parapsilosis* (CP1) were used in this study. The clinical *C. albicans* strains were donated by Professor Qingguo Qi of Shandong University of China. The isolates of *C. krusei*, *C. glabrata*, *C. tropicalis*, and *C. parapsilosis* were kindly provided by the Central Hospital of Jinan City. The two GFP-tagged *C. albicans* strains were previously constructed in our laboratory^28,40^. All isolates were stored in physiological saline supplemented with 20% glycerol at −80 °C and subcultured twice on YPD agar plates (2% tryptone, 1% yeast extract, 2% glucose and 2% agar) for 24 h at 30 °C. Prior to each experiment, the cells were cultured in liquid YPD medium (2% tryptone, 1% yeast extract, 2% glucose) overnight at 30 °C and 200 rpm.

Normal human bronchial epithelium (HBE) cells were donated from Professor Huiqing Yuan in Shandong University. They were grown in keratinocyte serum-free medium (Life Technologies, Inc. Grand Island, NY) on standard plastic ware (Falcon; Becton-Dickinson, Bedford, MA) at 37 °C in a 5% CO2 atmosphere as previously described^41^. The HeLa cell line was grown in Dulbecco’s Minimal Essential Medium (DMEM) supplemented with 10% fetal bovine serum (FBS), 1% L-glutamine and 1% antibiotic/antimycotic^42^.

### Chemicals

CA was previously isolated from two Chinese liverworts in our lab, and its structure was determined as previously reported (Fig. 1a)^17,18^. AMB was purchased from Sigma-Aldrich and used as a positive control in this study. Tu, NAC, SHAM, NaN_3_ and rotenone were purchased from Sigma.

### Minimum inhibitory concentration determination

The MIC_80_ of CA and AMB against *Candida* species were determined by the broth microdilution procedure following the CLSI M27-A3 guidelines^43^.

### Time-killing kinetics

To investigate the fungicidal effect of CA against *C. albicans*, time-killing curves were plotted by measuring viable cells in the presence of CA or AMB. *C. albicans* SC5314 cells were diluted to 1 × 10^6^ cells/ml in synthetic dextrose (SD) medium (0.67% yeast nitrogen base, 2% glucose, and amino acids) and exposed to different concentrations of CA or 4 μg/ml AMB at 30 °C. At different time intervals, an aliquot was removed and plated on YPD agar plates to enumerate surviving cells. All time-kill curve studies were conducted in duplicate.

### Effect of CA on *C. albicans* biofilm formation

*C. albicans* cells (1 × 10^6^ cells/ml in RPMI 1640 medium) were seeded into a 96-well tissue culture plate with final CA concentrations of 0, 8, 16, 32 and 64 μg/ml; 4 μg/ml AMB served as the positive control. Following incubation at 37 °C for 24 h, the supernatant was aspirated, and nonadherent cells were removed by washing with PBS. Then, the cells remaining in each well were quantified using an XTT reduction assay as previously described^22^.

### Effect of CA on pre-formed biofilms of *Candida* species

The wild-type *C. albicans* strain SC5314 and clinical isolate 28 A (a FLC-resistant isolate) and *C. tropicalis* CT3 (a FLC-resistant isolate) were used to evaluate the effect of CA against mature *Candida* biofilms. *Candida* biofilms were formed at 37 °C for 24 h on the surfaces of the wells of microtiter plates according to a previously reported method^44^. Briefly, *Candida* cells were suspended in RPMI 1640 at an initial density of 1 × 10^6^ cells/ml and transferred into the wells of microtiter plates. After incubation at 37 °C for 24 h, the supernatants were discarded, and nonadherent cells were removed by washing the biofilms three times with sterile PBS. To assess the effects of the antifungals on the pre-formed biofilms, CA or AMB was added, and the plates were incubated for an additional 24 h at 37 °C. The sessile cells were then removed from the microtiter wells by scraping the biofilms, vortexed vigorously to disaggregate clumps, 10-fold serially diluted with PBS, and plated on YPD agar plates to count surviving colonies.

For visual confirmation, GFP-tagged *C. albicans TDH3*-*GFP*-CAI4 was used in a live/dead two-color fluorescence assay^40^. The treated biofilms of *TDH3*-*GFP*-CAI4 were stained with 10 μg/ml PI for 1 h and washed three times with PBS. The stained samples were then observed using a Zeiss LSM 700 CLSM. In this assay of cell viability, healthy cells exhibit brilliant green fluorescence, whereas cells with damaged cell membranes stain fluorescent red due to intracellular accumulation of PI.

### *In vivo* antifungal test using *C. elegans* model

The antifungal activity of CA *in vivo* was evaluated using the *C. elegans*-*C. albicans* infection model as previously reported^45^. Briefly, *C. elegans* was pre-infected by wild-type strain SC5314 and challenged with different concentrations of CA. Addition of 1% DMSO served as the negative control. The survival state of infected worms was monitored daily and the survival rates were calculated. After 5 days of incubation, the nematodes were transferred to new wells and imaged using an Olympus microscope.

### Measurement of ROS generation

ROS generation was quantified by MitoSOX Red staining^46^. MitoSOX Red accumulates in the mitochondrial matrix and is oxidized to a fluorescent product by superoxide. *C. albicans* strain SC5314 (1 × 10^6^ cells/ml in SD medium) was incubated with increasing concentrations of CA or 4 μg/ml AMB at 30 °C for 3 h. Following staining with 20 µM MitoSOX Red for 30 min in the dark, the cells were collected, and the fluorescence intensity was measured by flow cytometry. The data were analyzed using WinMDI 2.9 software. Simultaneously, treated samples were stained with a combination of MitoSOX Red and SYTOX Green and visualized by a Zeiss LSM700 CSLM. SYTOX Green is excluded from living cells but fluoresces when intercalated in the DNA of dead cells^47^.

### Analysis of mitochondrial membrane potential (mtΔψ)

Rh123, a fluorescent stain that is distributed in the mitochondrial matrix directly in response to mtΔψ^48^, was used to investigate the effect of CA on the *C. albicans* mtΔψ in this study. SC5314 cells (1 × 10^6^ cells/ml in SD medium) were challenged with various doses of CA or 4 μg/ml AMB and incubated at 30 °C for 3 h. The samples were then stained with 20 μM Rh123 for 30 min in the dark and detected by flow cytometry^49^. The obtained data were analyzed by WinMDI 2.9 software.

### Measurement of intracellular ATP production

SC5314 cells (1 × 10^6^ cells/ml in SD medium) were challenged with various doses of CA or 4 μg/ml AMB and incubated at 30 °C for 3 h. Cells were then collected, washed with ice-cold PBS, and broken by the lysis buffer in the ATP Assay Kit (Beyotime Institute of Biotechnology, Haimen, China). 100 μl of each supernatant was mixed with 100 μl ATP detecting solution. The luminance at 560 nm emitted by the luciferase-mediated reaction of ATP and luciferin was measured by a Mithras LB940 reader (Berthold Biotechnologies, Bad Wildbad, Germany). Standard curves were also generated and the cellular protein concentrations were determined using a Bradford Protein Assay Kit (Beyotime Institute of Biotechnology, Haimen, China). The intracellular ATP level was calculated according to the standard curve and normalized using the protein contents of each sample and expressed as nmol/mg protein.

### Observation of the localization of Tom70-GFP

To assess the effect of CA on mitochondrial components, the mitochondrial outer membrane protein Tom70 was used. Specifically, Tom70 was labeled with GFP in the *C. albicans* CAI4 strain. The constructed strain, *TOM70-GFP-*CAI4 (1 × 10^6^ cells/ml), was treated with various doses of CA at 30 °C for 3 h in SD medium. The localization of Tom70 was visualized by CLSM with a 63 × objective lens using GFP as the indicator^28^.

### Effect of antioxidents and respiratory chain inhibitors on the antifungal activity of CA

*C. albicans* cells were diluted to 1 × 10^6^ cells/ml in SD medium and incubated with antioxidents (5 mM Tu or 5 mM NAC) or respiratory chain inhibitors (5 mM SHAM, 0.02% NaN_3_ or 10 μM rotenone) for 1 h at 30 °C, and followed with the treatment of 16 μg/ml CA. Cultures treated with CA alone served as control. After 3 h incubation, the viability of the cells was assessed by plating dilutions on YPD agar plates.

### Detection of plasma membrane dynamics

The plasma membrane of *C. albicans* cells was labeled by DPH to monitor cytoplasmic membrane dynamics in response to drug treatment according to a previously reported method^50^. SC5314 cells with an initial density of 1 × 10^6^ cells/ml in SD medium were incubated with CA or the positive control (4 μg/ml AMB) at 30 °C for 3 h. The cells were then fixed with 0.37% formaldehyde. After 30 min, the cells were collected, washed with PBS, and frozen in liquid nitrogen. For labeling, the cells were thawed and resuspended in PBS, followed by incubation with 0.6 mM DPH for 45 min at 28 °C. The fluorescence of the stained samples was measured in a spectrofluorophotometer (Berthold Biotechnologies, Bad Wildbad, Germany) with excitation at 350 nm and emission at 425 nm.

### Determination of membrane permeability

The change in membrane permeabilization in the presence of CA was assessed using the viable cell membrane-impermeable fluorescent dye PI. SC5314 cells (1 × 10^6^ cells/ml) were exposed to CA (0–64 μg/ml) or 4 μg/ml AMB in SD medium at 30 °C for 3 h and stained with 5 μg/ml PI for another 30 min in the dark. Then, the fluorescence intensity was measured by flow cytometry.

### Transmission electron microscopy (TEM)

The ultrastructure of *C. albicans* cells exposed to CA treatment was visualized by TEM. SC5314 cells were cultured in SD medium containing 32 μg/ml CA at 30 °C for 3 h and then harvested by centrifugation at 1000 × g for 5 min. Cells without drug treatment served as the control. The pellets were fixed, desiccated, and embedded as previously described^51^ before observation under a transmission electron microscope (JEM-1011, JEOL, Tokyo, Japan).

### Analysis of cell morphology

SC5314 cells (1 × 10^6^ cells/ml) were treated with various concentrations of CA in SD medium. After incubation for 3 h at 30 °C, the cells were harvested by centrifugation and suspended in PBS. The morphological changes were measured by flow cytometry (FACS Calibur; BD Biosciences, San Jose, CA) using FSC versus SSC two-dimensional contour plot to indicate changes in cell size and granularity, respectively^50^.

### Calcein leakage measurement

To reveal the effect of CA on cell membranes, we first established an artificial membrane using liposomes entrapping the fluorescent substance calcein according to a modification of a previously reported method^50^. We then measured the release of the fluorescent marker calcein from the liposomes upon treatment with CA. Specifically, a mixture of phosphatidylcholine/phosphatidylethanolamine/phosphatidylinositol/ergosterol (5:4:1:2,w/w/w/w) dissolved in chloroform was evaporated and subsequently dried under vacuum to form a dry thin membrane. The membrane was dispersed in a dye buffer solution [70 mM calcein, 10 mM Tris, 150 mM NaCl, and 0.1 mM EDTA (pH 7.4)] and mixed by vortexing to produce calcein-encapsulating multilamellar vesicles (MLVs). The suspension was freeze-thawed in liquid nitrogen for 11 cycles and then extruded through a polycarbonate filter (100-nm pore size filter, 11 times) using an Avanti-mini extruder (Avanti Polar Lipids, Alabaster, USA). Untrapped calcein was removed by gel chromatography on a Sephadex G-50 column.

The prepared calcein-encapsulating LUVs were treated with the indicated concentration of CA or AMB for 3 h. Calcein release was quantified by measuring the fluorescence intensity (λex = 490 nm, λem = 520 nm) with a Tecan Spark 10 M microplate reader. Triton X-100 was used to release the dye completely from the vesicles (100% dye leakage). The percentage of dye leakage caused by CA or AMB was calculated as follows: % dye leakage = 100 × (F − F_0_)/(F_t_ − F_0_), where F represents the fluorescence intensity after the addition of the compounds and F_0_ and F_t_ represent the fluorescence intensities without the compounds and with Triton X-100, respectively^50^.

### The cytotoxicity of CA

3-(4,5-Dimethylthiazol-2-yl)-2,5-diphenyl-2H-tetrazoliumbromide (MTT, Sigma) colorimetric assay was used to quantitate normal HBE and HeLa cell proliferation and cytotoxicity in the presence of CA. Cells (1 × 10^4^ per well) were seeded into 96-well plates as previously described culture conditions^41,42^. After 24 hour incubation, the cells were treated with vehicle, or desired concentrations of CA for further 48 h. After removing the medium, cells were incubated with 10 μl of MTT for 4 h. The cell growth response to the chemicals was detected by measuring the light absorbance at 570 nm on a plate reader (Bio-Rad Laboratories, Richmond, CA).

In addition, we utilized cell membrane impermeable fluorescent dye PI and ROS fluorescent probe DCFHDA separately to determine the permeability and intracellular ROS contents under the treatment of CA. Hela cells were cultured for 24 h, serial concentrations of CA were added into cells. After 12 h treatment, cells were collected and stained with 5 μg/ml of PI or 5 μM DCFH-DA for 30 min in the dark. Flow cytometry (FACS Calibur; BD Biosciences, San Jose, CA) was then used to measure the fluorescence intensity. The red fluorescence of PI and green fluorescence of DCF was excited by a 488-nm laser and collected in the FL2 channel and FL1 channel, respectively. A total of 10,000 events in each sample were recorded for analysis. The resultant data were processed using WinMDI 2.9 software (Joseph Trotter, The Scripps Institute, La Jolla, CA).

### Statistical analysis

All experiments were performed in triplicate in different days. The results were represented as the means ± SDs. The nematodes data were analyzed by Kaplan-Meier method using SPSS Statistical Package 19.0. Other experimental data were statistically analyzed by two-tailed Student’s t-test and a *P* value < 0.05 indicated statistical significance.
